# Exome sequencing in pooled DNA samples to identify maternal pre-eclampsia risk variants

**DOI:** 10.1038/srep29085

**Published:** 2016-07-07

**Authors:** Tea Kaartokallio, Jingwen Wang, Seppo Heinonen, Eero Kajantie, Katja Kivinen, Anneli Pouta, Paul Gerdhem, Hong Jiao, Juha Kere, Hannele Laivuori

**Affiliations:** 1 Medical and Clinical Genetics, University of Helsinki and Helsinki University Hospital, Helsinki, Finland; 2 Department of Biosciences and Nutrition, and Science for Life Laboratory, Karolinska Institutet, SE-141 83 Stockholm, Sweden; 3 Obstetrics and Gynaecology, University of Helsinki and Helsinki University Hospital, Helsinki, Finland; 4 Chronic Disease Prevention Unit, National Institute for Health and Welfare, Helsinki, Finland; 5 Children’s Hospital, Helsinki University Hospital and University of Helsinki, Helsinki, Finland; 6 PEDEGO Research Unit, MRC Oulu, Oulu University Hospital and University of Oulu, Oulu, Finland; 7 Division of Cardiovascular Medicine, University of Cambridge, Cambridge, UK; 8 Department of Government services, National Institute for Health and Welfare, Helsinki, Finland; 9 Department of Orthopedics, Karolinska University Hospital, Stockholm, Sweden; 10 Department of Clinical Sciences, Intervention and Technology (CLINTEC), Karolinska Institutet, SE-141 86 Stockholm, Sweden; 11 Molecular Neurology Research Program, University of Helsinki, Helsinki, Finland; 12 Folkhälsan Institute of Genetics, Helsinki, Finland; 13 Institute for Molecular Medicine Finland, University of Helsinki, Helsinki, Finland

## Abstract

Pre-eclampsia is a common pregnancy disorder that is a major cause for maternal and perinatal mortality and morbidity. Variants predisposing to pre-eclampsia might be under negative evolutionary selection that is likely to keep their population frequencies low. We exome sequenced samples from a hundred Finnish pre-eclamptic women in pools of ten to screen for low-frequency, large-effect risk variants for pre-eclampsia. After filtering and additional genotyping steps, we selected 28 low-frequency missense, nonsense and splice site variants that were enriched in the pre-eclampsia pools compared to reference data, and genotyped the variants in 1353 pre-eclamptic and 699 non-pre-eclamptic women to test the association of them with pre-eclampsia and quantitative traits relevant for the disease. Genotypes from the SISu project (n = 6118 exome sequenced Finnish samples) were included in the binary trait association analysis as a population reference to increase statistical power. In these analyses, none of the variants tested reached genome-wide significance. In conclusion, the genetic risk for pre-eclampsia is likely complex even in a population isolate like Finland, and larger sample sizes will be necessary to detect risk variants.

Pre-eclampsia is a common and complex vascular pregnancy disorder. It is characterized by hypertension and proteinuria, and often involves impaired placental development^1^,^2^. The disease is a major cause for both maternal and perinatal mortality and morbidity^3^, and predicts increased risk of chronic cardiometabolic diseases later in life^4^,^5^.

Genetic factors contribute to pre-eclampsia susceptibility. Heritability estimates for pre-eclampsia range between 0.54 and 0.68, consisting of both maternal and fetal contribution^6^,^7^. Risk for pre-eclampsia is elevated after first pre-eclamptic pregnancy^8^, and in women with an affected first-degree relative^9^. Genome-wide linkage studies in pre-eclampsia families have revealed several susceptibility loci for the disease^10^,^11^,^12^,^13^,^14^. Also, numerous candidate gene studies (summarized in meta-analyses^15^,^16^,^17^), mostly assessing the effect of maternal genotype, and two relatively modestly sized genome-wide association studies on maternal pre-eclampsia risk have been published^18^,^19^. Despite these attempts to discover genetic risk variants for pre-eclampsia, no robustly replicated candidate genes have yet been identified.

Because pre-eclampsia is a major cause for both maternal and fetal mortality, preterm birth and fetal growth restriction^3^,^20^,^21^, it can be assumed to reduce reproductive success. Therefore, negative evolutionary selection likely maintains population frequencies of pre-eclampsia risk variants low. Following this reasoning, we focus on screening for low-frequency (minor allele frequency (MAF) 1–5%) maternal variants with large or moderate effect on the risk of pre-eclampsia.

The population history of Finland is characterized by strong founder effect, periods of rapid population growth, and internal migrations and establishment of population isolates as late as in the 16^th^ century (summarized in refs 22,23). The Finnish population has therefore gone through multiple relatively recent bottlenecks, and evolutionary selection has not eliminated deleterious variants as effectively as in older and more outbred populations. This has led to enrichment of low-frequency loss-of-function variants^24^. Number of samples required to reach adequate statistical power to detect associations with some pathogenic variants might therefore be reduced in the Finnish population^25^, making it an ideal choice for studies concentrating on low-frequency variants.

Although the cost of next-generation sequencing has reduced considerably over the past years, costs for studies utilizing these methods still remain substantially high. Pooling DNA samples together can significantly decrease the cost of genome-wide sequencing, especially in the studies targeting rare or low-frequency variants^26^. Here we exploited this idea by pooling DNA from a hundred pre-eclamptic women in pools of ten, and by exome sequencing the pools to screen for pre-eclampsia risk variants.

## Samples and Methods

### Study participants

The sample set utilised in the exome sequencing included a hundred Finnish pre-eclamptic women, pooled in pools of ten. Ninety of the study participants were selected from the Finnish Genetics of Pre-eclampsia Consortium (FINNPEC) case-control cohort that was collected from five Finnish university hospitals during 2008 to 2011^27^. Ten participants belong to separate families chosen from the pre-eclampsia family cohort recruited from the Kainuu and Helsinki regions^10^. All the FINNPEC cases included in the exome sequencing had severe pre-eclampsia. Two of the pools contained women with early-onset disease, three pools women with high proteinuria, one pool women with previous miscarriages, and one pool women with recurrent pre-eclampsia (Supplementary Table S1). Women with severe pre-eclampsia were prioritised in the sample selection as they are more likely to possess genetic risk factors for the disease. Clinical definitions as well as the sample collection and DNA extraction methods are described in Supplementary File 1.

The first individually Sequenom genotyped sample consisted of 180 pre-eclamptic and 180 healthy pregnant women, including the 100 pre-eclamptic cases originally exome sequenced, and additional study subjects from the FINNPEC cohort. The exclusion criteria for the controls were any pregnancy complication in the current pregnancy, pre-eclampsia in any pregnancy, chronic hypertension and pregestational diabetes mellitus.

The second individually genotyped sample set included 1353 pre-eclamptic and 699 non-pre-eclamptic women. These also include all the samples from the first Sequenom genotyping, all of which except the family samples were re-genotyped in the second run. All the women, except for the ten family cohort members, were selected from the FINNPEC cohort. The inclusion criterion for the cases was pre-eclampsia, and for the controls non-pre-eclamptic pregnancy. The exclusion criteria for the controls were pre-eclampsia in any pregnancy, small-for-gestational age (SGA) infant or placental insufficiency, gestational hypertension, chronic hypertension and placental ablation.

All study participants have provided a written informed consent. The study protocols have been approved by the Coordinating Ethics Committee of the Hospital District of Helsinki and Uusimaa, and the methods were carried out in accordance with the approved guidelines.

Variant data from a hundred Swedish scoliosis cases^28^ that were exome sequenced with a pooled strategy identical to ours were utilised in the exome sequencing variant filtering to omit false positive and common variants.

The Sequencing Initiative Suomi (SISu 3.0) data set was utilised in the Sequenom phase 2 association analysis as population specific reference data to increase the statistical power. The SISu database is available online ( http://www.sisuproject.fi/) and at the time of the analysis contained exome sequence data from over 6100 Finns. The data set was utilized as a reference unselected for the pre-eclampsia phenotype as this information was not available for the sequenced individuals.

A summary of the study participants is shown in Table 1.

### Exome sequencing

Details of the exome sequencing are described in Supplementary File 1. The initial quality control of the sequencing data was performed by the sequencing service provider (Science for Life Laboratory, Stockholm, Sweden). The paired-end reads were aligned to the human reference genome hg19 with the Burrows-Wheeler Aligner (BWA) software package, version 0.6.1^29^. SAMtools version 0.1.18^30^ was used to remove PCR duplicates in each pool, to filter out multiply mapped reads (mapping quality score <20) and to call variants. The uniquely mapped reads were used as input for variant (SNV) calling for each pool with the SAMtools default setting, and the variants identified were merged together. Annotation was completed using the ANNOVAR software^31^ with dbSNP version 137 and the 1000 Genomes 2012 APR^32^. BEDtools, version 2.16.2^33^ was applied for evaluating read depth and coverage.

The following filtering criteria were applied to the variants called. The missense, nonsense and splice site variants that were present in at least two pre-eclampsia pools were considered further. Insertions and deletions were excluded from the analysis, because they could not be called reliably. Exome sequencing data from Swedish scoliosis patients were produced with a pooling strategy identical to ours and were utilised in the filtering as a technical control to exclude false positive and common variants. The variants present in over five scoliosis pools were excluded. The variants present in less than two scoliosis pools were filtered for European allele frequency in the 1000 Genomes data, and only the variants with MAF ≤0.05 were included. MAF of each variant in the exome-sequenced samples was estimated by calculating the proportion of reads carrying minor allele in the total amount of reads covering the position. The MAF estimation is based on an assumption that each sample is equally represented within a pool. The MAF estimates in the pre-eclampsia pools were compared to the MAFs in the reference data sets (SISu 2014, the 1000 Genomes European data APR2012 and the scoliosis exome sequencing data). By using three reference data sets we were able to obtain comprehensive picture of the variant frequencies in the Finnish and European populations, and also to estimate relevance of those variants that were absent in the population-specific reference data SISu. The variants with pre-eclampsia_MAF/reference_MAF ratio ≥1.5 in all the available comparisons were selected. In addition, five variants that were close to the cut-off or had high ratio in one or two of the comparisons were included. The following procedures were also utilised. Variation located in the genes listed in the papers by Fuentes Fajardo *et al.*^34^ and Ju *et al.*^35^ were excluded. These papers have listed genes that contain excess amount of variation, likely due to assembly errors in the reference sequence or alignment errors e.g. in highly polymorphic genomic regions. The variants flagged “suspected” in the dbSNP database were excluded. For the variants with no MAF information available in our default reference data sets, we utilised data from other sources (the 1000 Genomes ALL, CSAgilent), and excluded the variants with MAF higher than or similar to the MAF in our data. In addition to the main filtering strategy, we also focused on the linkage peak regions identified in the previous studies by our group^10^,^36^, and selected the linkage peak region variants that were present in the pool containing pre-eclampsia family samples, had the 1000 Genomes EUR MAF ≤0.05, and pre-eclampsia_MAF/reference_MAF≥1.5. We also looked for candidate variants among the SNPs whose reference allele was the minor allele. For this filtering strategy we selected the missense, nonsense and splice site variants with the 1000 Genomes European reference allele frequency ≤0.05, and pre-eclampsia_refAllele/SISu and pre-eclampsia_refAllele/scoliosis_refAllele ≥1.5, and selected the variants whose reference allele was present in ≤2 scoliosis pools and in over two pre-eclampsia pools.

### Sequenom genotyping

Two rounds of Sequenom genotyping were performed. The purpose of the first round was to verify the presence and enrichment of the selected variants individually in the original exome-sequenced samples, whereas the second round was conducted to test the association of the variants with pre-eclampsia.

Of the 59 variants selected based on the exome sequencing, 46 were directly fitted to three Sequenom iplexes. In addition, two of the variants were captured through tagging SNPs. Rs117741116 tagged rs139702277 with r^2^ = 1 and D′ = 1 in the 1000 Genomes FIN and CEU populations, and rs12462506 tagged rs3745601 with r^2^ = 0.96 and D′ = 1 in the 1000 Genomes FIN population. The rest of the SNPs were left out, because of issues in assay design. In total, 48 variants were genotyped. The first, the second and the third iplexes contained 24, 17 and 7 variants, respectively. For the second genotyping run, 28 variants were selected for genotyping in two iplexes. In addition, rs6025 in *F5* was included to the panel to investigate if the previously found association between the variant and pre-eclampsia^15^,^16^,^17^ would be replicated in our data. The assay design and the genotyping were performed with Sequenom MassArray system at the Institute for Molecular Medicine Finland FIMM Technology Centre, University of Helsinki. The Technology Centre performed routine quality control steps to ensure high quality of the genotyping.

### Association analysis

Association of the variants with pre-eclampsia was evaluated by chi-square test in the PLINK software v1.07^37^. Genotypes from the Finnish SISu (3.0) data set containing 6118 individuals were combined with our control data to increase the statistical power. Association test was conducted both in the FINNPEC case-control data set and in the merged FINNPEC – SISu data. Two X chromosomal variants were excluded from the latter analysis, because the SISu data contain also males. The allele frequencies of autosomal variants are not assumed to differ between females and males. Any Sequenom genotyped sample with failed genotyping for >2 variants that had been otherwise successfully genotyped was removed from the analysis. Hardy-Weinberg equilibrium (HWE) was calculated independently for the Sequenom genotyped case-control samples, for the SISu data and for the merged FINNPEC-SISu data. Differential genotype missingness between the cases and controls was tested in the FINNPEC cohort.

A standard linear regression model in the PLINK software was applied for testing association of the genetic variants with quantitative traits relevant for pre-eclampsia in the FINNPEC cohort. The quantitative traits utilized in the association testing included the highest systolic blood pressure, the highest diastolic blood pressure, proteinuria and relative birth weight of the baby. An additive model was applied in the analyses.

Statistical power was calculated with Genetic Power Calculator using the “case-control for discrete traits” module^38^ ( http://pngu.mgh.harvard.edu/~purcell/gpc/cc2.html). With a pre-eclampsia prevalence of 0.05 and a risk allele frequency of 0.05, our cohort of 1353 pre-eclamptics and ~6800 controls (the non-pre-eclamptic FINNPEC controls and the SISu (3.0) data unselected for the pre-eclampsia phenotype combined), was estimated to be sufficient to detect an effect size of 1.65 for risk heterozygote and 3 for risk homozygote with power of 0.80 when α < 5 × 10^−8^. Under the aforementioned parameters, for the variants with a risk allele frequency of 0.01, we could detect effect sizes of 2.52 and 5 for the risk heterozygote and homozygote, respectively.

## Results

### Clinical characteristics

Clinical characteristics of the study participants are presented in Table 2. The pre-eclamptic women in both Sequenom sample sets delivered on average earlier and had babies with lower absolute and relative birth weight than the control women. In the second Sequenom genotyping sample set the number of primiparous women was larger and BMI higher among the pre-eclamptic women, and larger percentage of them was affected by pregestational or gestational diabetes.

### Exome sequencing data

**Figure 1:**
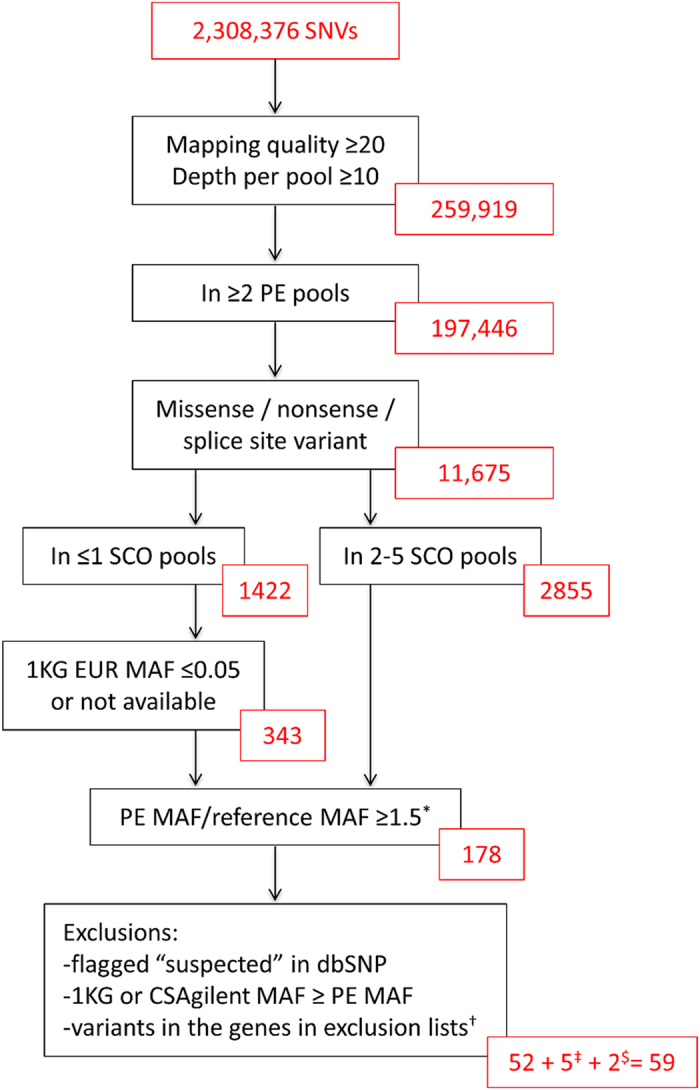
The main filtering strategy for the variants identified in the exome sequencing. The variants that passed quality criteria and were found in at least two pre-eclampsia pools were filtered for function. To further narrow down the number of variants, pooled exome sequencing data from Swedish scoliosis patients as well as the 1000 Genomes European data and the Finnish Sequencing Initiative Suomi (SISu) data were utilised in the filtering. Variants remaining after each filtering step are indicated in red boxes. Based on the main filtering steps, 52 variants were selected. Furthermore, seven variants were selected based on additional filtering strategies. ^*^The reference data used: the pooled exome sequencing data from Swedish scoliosis patients; the 1000 Genomes European data (APR 2012); and the Finnish SISU data (2014). ^†^Fuentes Fajardo *et al.*^34^ and Ju *et al.*^35^. ^‡^The missense, nonsense and splice site variants with the 1000 Genomes European reference allele frequency ≤0.05, pre-eclampsia_refAllele/SISu and pre-eclampsia_refAllele/scoliosis_refAllele ≥1.5, reference allele present in ≤2 scoliosis pools and in over two pre-eclampsia pools. ^$^A nonsense variant present in only one pre-eclampsia pool and a variant located in the previously identified linkage peak region were selected outside the main filtering strategy. 1 KG = The 1000 Genomes Project; MAF = minor allele frequency; PE = pre-eclampsia, SCO = the pooled exome sequencing data from Swedish scoliosis patients.

The sequencing provider delivered a total number of 299 to 482 million reads in each sample pool. Over 98% of the reads could be mapped to the reference genome hg19. Per pool on average, 90% and 80% of the SureSelect target regions were covered by a depth of at least 30x and 60x, respectively, and the average depth of the target regions per pool was between 95x and 277x, the average of all pools being 220x. Eight of the ten pools had average coverage over 200x in the enrichment regions. The total number of variants called was 2,308,376. The main filtering strategy applied to these variants is illustrated in Fig. 1 and explained in detail in the Samples and Methods section. After excluding the variants with mapping quality <20 or depth <10x, there were 259,919 variants left, of which 31,579 were located in protein-coding regions. By applying the filtering steps, we identified 59 candidate variants that seemed to be enriched in the exome sequenced pre-eclamptic women (Supplementary Table S2).

### The first round of Sequenom genotyping in the FINNPEC samples

The first round of Sequenom genotyping was carried out in a sample set of 180 cases and 180 controls including the 100 exome sequenced samples. The purpose of this step was to verify the presence of the variants in the original samples and the MAF difference between pre-eclamptic women and general population. All assays had a success rate >95%. Of the 48 variants that were genotyped, three were monomorphic. After excluding these variants, correlation coefficient between the MAF estimates from the exome sequencing and the MAFs obtained from the Sequenom genotyping for the exome sequenced samples was 0.94, showing that we were able to estimate MAFs in the exome sequencing fairly accurately. Four of the variants were not in HWE in the controls. Twenty-eight variants that had OR ≤0.65 or ≥1.3 were selected for the second round of Sequenom genotyping in a larger case-control cohort. The results of the first Sequenom round are shown in Supplementary Table S3.

### The second round of Sequenom genotyping in the FINNPEC samples

In the second round of genotyping all the variants except failed rs6681 were successfully genotyped. After excluding 17 cases and 5 controls with more than 2 failed genotypes, the genotyping rate for the variants in the remaining individuals was over 96%. Concordance rate of the genotypes in the 350 individuals included in both Sequenom runs was 0.9992. Three individuals with at least one discordant genotype between the runs were excluded. Association of the variants with pre-eclampsia was first tested in the Sequenom genotyped FINNPEC case-control sample set. Three variants deviated from HWE in the FINNPEC controls and were omitted. In the association analysis, one of the genotyped variants was nominally associated with pre-eclampsia (rs79744308 in *NRTN*; p-value = 0.0314, OR (95% CI) = 0.69 (0.48–0.97)), but the variant did not pass differential missingness test between the cases and controls. None of the association tests reached genome-wide significance. Full results are shown in Supplementary Table S4.

### Association analysis in the combined FINNPEC-SISu data set

In order to increase statistical power, we merged the FINNPEC data with the Finnish SISu (3.0) data, and tested association of the genotyped variants with pre-eclampsia in this combined data set. Three of the variants deviated from HWE in the controls in these data, and were excluded from the analysis. Furthermore, the X chromosomal variants were excluded as the SISu data contain both males and females. In the association analysis in the merged data, we detected nominal association of four variants (Table 3). None of them, however, reached genome-wide significance. Full results from this analysis are presented in Supplementary Table S4. Comparison of MAFs between different sample subsets for the 28 variants genotyped in the second Sequenom genotyping is shown in Supplementary Table S5.

The variant rs6025, which is located in the *F5* gene and has previously been connected to pre-eclampsia^15^,^16^,^17^, was not associated with pre-eclampsia in our study in either of the analyses.

### Quantitative traits association analysis

Assuming an additive model of genetic inheritance, we investigated the association between the genotyped SNPs and quantitative clinical characteristics of pre-eclampsia (Supplementary Table S6). Among the SNPs nominally associated with pre-eclampsia (Table 3), rs3803339/G allele in *TP53BP1* showed nominal association (p-value = 0.026) with the highest systolic blood pressure, and rs61747120/T in *ZFR2* with proteinuria (p-value = 0.039), both in the pre-eclamptic women. In addition to those two SNPs, there were several other variants associated with the highest systolic or diastolic blood pressure, relative birth weight of the baby or proteinuria in the pre-eclamptic patients, the non-pre-eclamptic controls or the whole case-control sample set (Supplementary Table S6). Rs2291516 in *RGL3* was associated with both proteinuria (p-value = 0.001) and relative birth weight of the baby (p-value = 7.3 × 10^−4^). However, none of the SNPs were associated with the quantitative traits at the genome-wide significance level.

## Discussion

In this first attempt to screen exome-wide for low-frequency, moderate or large-effect risk variants for pre-eclampsia in a Finnish founder population, we did not find any genome-wide significantly associated risk variants. Whereas many complex diseases such as type 2 diabetes or cardiovascular diseases often have onset at midlife or later, pre-eclampsia affects women at their reproductive years, or if the phenotype of offspring is considered, already during the fetal period. Consequently, the disease decreases reproductive success, and there is a reason to hypothesise that pre-eclampsia risk variants are under negative evolutionary selection, which would keep their population frequencies low. A couple of GWA studies have assessed the role of common variation in pre-eclampsia susceptibility^18^,^19^, but low-frequency variation has not previously been studied exome- or genome-wide. A founder population such as Finland, which has gone through relatively recent bottlenecks and is enriched for low-frequency loss-of-function mutations^24^, should be an ideal study population for this screening.

The DNA samples were exome sequenced in pools of ten in order to maximise the number of sequenced samples while maintaining the sequencing costs low. With this strategy we were able to perform cost-effective exome-wide screen for pre-eclampsia risk variants, but the approach also has several limitations, such as missing information on individual genotypes and on exact MAF of each variant in the study sample. We were able to estimate MAFs from the pooled exome-sequenced samples fairly accurately: correlation coefficient between our estimates and the 1000 Genomes European data was 0.972 when the sequencing depth was at least 30x per pool. We however acknowledge that variants with biased MAF estimates were prone to be selected for the Sequenom genotyping, whereas some truly enriched variants could have been missed. In the association analysis we utilized the large SISu data as a population specific reference to increase statistical power of the analysis. Both the FINNPEC data and the data sets contributing to the SISu project have been collected to represent the Finnish population. However, population substructure within Finland^39^,^40^ could cause bias especially when studying low-frequency variants. For some of the variants allele frequencies differ between the FINNPEC and SISu controls indicating potential for subtle population stratification. We were unable to test the existence of population stratification due to the limited number of SNPs genotyped in our data. Another potential source of bias is that the data sets have been genotyped with different methods.

There are several possible explanations for not identifying pre-eclampsia risk variants in this study. Our screening did not cover non-coding regions, and also structural variation was omitted from the analysis. Furthermore, variants in low-coverage regions could have been missed. At the time of the exome sequencing data analysis robust variant callers with ploidy setting were not available. Utilizing a caller assuming a diploid genome might have caused us to miss some candidate variants. Some risk variants may have been lost due to choices made in the filtering. As shown previously, variant annotations are much dependent on a transcript set and annotation software utilized, and often there are several plausible annotations for a single variant^41^. Variants with incorrect or alternative annotations may have been unintentionally filtered out. Genetic risk for pre-eclampsia might be heterogeneous even in an isolated population, and a larger sample size might have been needed to find predisposing variants. As the sample size in the exome sequencing was modest, false negative results can occur. Furthermore, our study was underpowered to detect rare variants or variants with small effect sizes. Especially in the case of rare pre-eclampsia risk variants, gene-based testing methods might be needed to reveal disease association. The approach taken in this study was nevertheless justified. In another study we and co-workers used identical design to reveal genetic variants associated with morbid obesity, and identified a low-frequency variant that showed strong association with BMI^42^, a complex trait that is similarly to pre-eclampsia affected by multiple genetic and environmental factors.

Although we could not detect any robust risk variant for pre-eclampsia in this study, we mention here five missense variants nominally associated either with the disease phenotype (rs3803339 in *TP53BP1,* rs61747120 in *ZFR2*, rs113926353 in *ANO9*, and rs142394560 in *TMTC1*), or with proteinuria and relative birth weight of the baby in the pre-eclamptic cases (rs2291516 in *RGL3*). These variants or genes have not previously been linked to pre-eclampsia or any other pregnancy disorders. *TP53BP1* encodes a protein involved in DNA damage response and cell cycle regulation^43^. Of interest, variants in *TP53BP1* have shown a suggestive association with blood pressure^44^. ZFR2 is a zinc finger RNA binding protein with an unknown function. ANO9 belongs to a family of calcium-dependent chloride channels, and might suppress baseline chloride conductance^45^,^46^, and TMTC1 is an endoplasmic reticulum protein involved in calcium homeostasis^47^. RGL3, which interacts with Rap-family G-proteins^48^, has been shown to affect cell growth and morphology^48^,^49^. *TP53BP1*, *TMTC1* and *RGL3*^48^ are expressed in a wide range of tissues, *ANO9* most abundantly in skin and digestive tract and *ZFR2* in adrenal gland, cerebral cortex and testis ( http://www.proteinatlas.org/). A larger sample size would have been needed to state anything conclusive about the role of the variants in these genes in the risk of pre-eclampsia.

Rs6025 located in the *F5* gene has been associated with pre-eclampsia in tens of candidate gene studies (summarized in meta-analyses^15^,^16^,^17^), and was therefore included as an additional variant in the Sequenom panel. *F5* encodes the Factor V protein, a central component in the coagulation pathway. The amino acid change produced by rs6025 prevents inactivation of Factor V, which increases tendency to thrombosis^50^. To the best of our knowledge this is one of the largest original studies to investigate connection between rs6025 and pre-eclampsia. In contrast to many previous studies, our study does not provide support for the association of this variant with pre-eclampsia.

Along with two published GWA studies, this study is one of the firsts to screen for pre-eclampsia risk variants exome- or genome-wide. In future studies, a hypothesis-free design with larger sample sizes is required, as shown for many other complex phenotypes. One of the features of pre-eclampsia is the involvement of two individuals: a mother and a child. Therefore, the genetic information of children should be included in studies on pre-eclampsia, and interaction between fetal and maternal genotypes should be studied more comprehensively. The multinational InterPregGen consortium has addressed the need for larger sample sizes and for studying maternal and fetal genotypic interaction by GWAS genotyping 7600 pre-eclamptic mothers, 4000 pre-eclamptic infants and 46000 control women with the aim of identifying genetic risk factors for pre-eclampsia^51^. The FINNPEC cohort is involved as a replication cohort in this biggest effort in genetics of pre-eclampsia to date. In parallel with case-control studies on sporadic pre-eclampsia, studies in pre-eclampsia families may help to identify genes and pathways involved in the disease susceptibility. We have taken this approach and are currently screening for risk variants in the Finnish pre-eclampsia families with next-generation sequencing methods.

In this first exome-wide screening for pre-eclampsia risk variants we did not find any variant that would have been robustly associated with the disease phenotype. We conclude that even in a population isolate like Finland, the genetic risk for pre-eclampsia is likely complex and heterogeneous, and genome-wide approaches with larger sample sizes will be necessary to detect risk factors.

## Additional Information

**How to cite this article**: Kaartokallio, T. *et al.* Exome sequencing in pooled DNA samples to identify maternal pre-eclampsia risk variants. *Sci. Rep.* **6**, 29085; doi: 10.1038/srep29085 (2016).

## Supplementary Material

Supplementary Information

Supplementary Table S1

Supplementary Table S2

Supplementary Table S3

Supplementary Table S4

Supplementary Table S5

## Figures and Tables

**Table 1:** Summary of the study participants utilised in the exome sequencing and in the association analysis.

	Exome sequencing		Association analysis		
	PE cases	Scoliosis reference for filtering*	PE cases	Non-PE controls	SISu reference
Sample size	100	100	1353	699	6118
Female%	100%	76%	100%	100%	57%
Ancestry	Finnish	Swedish	Finnish	Finnish	Finnish

^*^Swedish scoliosis patients that were pooled and exome sequenced with the same method as the pre-eclamptic cases. These controls were utilised as a technical control to filter out false positive and common variants. PE = pre-eclampsia; SISu = Sequencing Initiative Suomi.

**Table 2:** Clinical characteristics of the study participants.

Maternal or perinatal characteristics	Exome-sequencing	Sequenom genotyping 1			Sequenom genotyping 2		
	Pre-eclampsia(n = 100)	Pre-eclampsia(n = 180)	Control(n = 180)	P	Pre-eclampsia(n = 1353)	Control(n = 699)	P
Age (years)	30 (27/32)	29 (26/32)	28 (25/32)	0.515	30 (26/34) [1352]	29 (26/33)	0.010
Primipara	89 (89.0)	166 (92.2)	162 (90.0)	0.459	999 (73.8)	379 (54.2)	<0.001
BMI (kg/m^2^)	22.7 (20.8/25.1) [94]	22.4 (20.4/25.2) [174]	22.8 (20.7/25.0)	0.685	24.0 (21.3/27.8) [1344]	23.0 (20.8/25.8)	<0.001
Highest systolic blood pressure (mmHg)	168 (161/181)	168 (160/179)	125 (118/133)	<0.001	165 (154/179)	125 (118/133)	<0.001
Highest diastolic blood pressure (mmHg)	111 (105/117) [99]	111 (106/116) [179]	83 (78/88)	<0.001	109 (104/116) [1352]	82 (78/87)	<0.001
Proteinuria (g/24 h)	4.5 (2.3/7.3) [97]	4.8 (2.7/7.2) [177]	NA	NA	3.1 (1.3/6.1) [1202]	0.9 (0.7/1.3) [6]	NA
Birth weight (g)	2650 (2006/3172)	2628 (1943/3123)	3543 (3256/3909)	<0.001	2770 (2075/3300)	3630 (3312/3950)	<0.001
Relative birth weight (SD)	−1.29 (−1.88/−0.48) [99]	−1.38 (−1.95/−0.52) [179]	−0.12 (−0.68/0.55)	<0.001	−1.13 (−1.88/−0.30) [1351]	0.02 (−0.58/0.69) [698]	<0.001
Gestational age at birth (weeks)	37.4 (34.4/39.0) [99]	37.4 (34.6/38.9) [179]	40.6 (39.7/41.3)	<0.001	37.7 (35.1/39.1) [1352]	40.4 (39.4/41.3)	<0.001
Chronic hypertension	3 (3.0) [99]	3 (1.7) [179]	0	0.123	235 (17.4) [1352]	0	<0.001
Pregestational diabetes mellitus	0 [99]	0 [179]	0	NA	40 (3.0) [1352]	5 (0.7)	0.001
Gestational diabetes mellitus	1 (1.0) [99]	1 (0.6) [179]	0	0.499	184 (13.6) [1352]	53 (7.6)	<0.001
Smoking before pregnancy	13 (14.4) [90]	30 (17.6) [170]	33 (19.0) [174]	0.752	222 (17.4) [1277]	137 (20.4) [670]	0.098
Smoking during pregnancy	6 (6.5) [92]	10 (5.8) [172]	11 (6.3) [176]	0.864	84 (6.5) [1289]	56 (8.2) [682]	0.164

Values for continuous variables are median (25^th^/75^th^ percentile), and for categorical variables frequencies (% in parentheses). Number of study participants is shown in brackets if different. Comparison of continuous variables was performed with the Mann–Whitney U test due to non-normality of the data. Categorical variables were analysed using the chi^2^ test or the Fisher’s exact test if number of observations in any cell was <5. PE = pre-eclampsia; BMI = body mass index.

**Table 3:** The SNPs nominally associated with pre-eclampsia in the analysis in the combined FINNPEC and SISu data set.

Gene	rs number	A2	MAF PE case	MAF non-PE ctrl	MAF SISu	MAF SISu + non-PE ctrl	P*	OR (CI 95%)*
*TP53BP1*	rs3803339	G	0.046	0.038	0.035	0.035	0.0058	1.33 (1.09–0.63)
*ZFR2*	rs61747120	T	0.066	0.067	0.054	0.055	0.0237	1.22 (1.03–1.44)
*ANO9*	rs113926353	G	0.036	0.038	0.045	0.044	0.0477	0.80 (0.64–1.00)
*TMTC1*	rs142394560	A	0.040	0.031	0.032	0.032	0.0477	1.25 (1.00–1.57)

^*^Pre-eclamptic cases compared to the combined control data (the non-pre-eclamptic FINNPEC controls + the SISu (3.0) data, in which information on pre-eclampsia was not available). A2 = minor allele; MAF = minor allele frequency; PE = pre-eclampsia; SISu = Sequencing Initiative Suomi; OR = odds ratio; CI = confidence interval.
