# Supplementary material for: Exome sequencing in pooled DNA samples to identify maternal pre-eclampsia risk variants
Source: Sci Rep. 2016 Jul 7;6:29085. doi: 10.1038/srep29085 (PMC4935848; doi:10.1038/srep29085)
Supplement: Supplementary Information [file srep29085-s1.pdf]

Exome sequencing in pooled DNA samples to identify maternal pre-eclampsia risk variants

Tea Kaartokallio[1†*], Jingwen Wang[2†], Seppo Heinonen[3], Eero Kajantie[4,5,6], Katja Kivinen[7], Anneli Pouta[6,8], Paul Gerdhem[9,10], Hong Jiao[2], Juha Kere[2,11,12], Hannele Laivuori[1,3,13]

1 Medical and Clinical Genetics, University of Helsinki and Helsinki University Hospital, Helsinki, Finland

2 Department of Biosciences and Nutrition, and Science for Life Laboratory, Karolinska Institutet, Stockholm, Sweden

3 Obstetrics and Gynaecology, University of Helsinki and Helsinki University Hospital, Helsinki, Finland

4 Chronic Disease Prevention Unit, National Institute for Health and Welfare, Helsinki, Finland

5 Children's Hospital, Helsinki University Hospital and University of Helsinki, Helsinki, Finland

6 PEDEGO Research Unit, MRC Oulu, Oulu University Hospital and University of Oulu, Oulu, Finland

7 Division of Cardiovascular Medicine, University of Cambridge, Cambridge, UK

8 Department of Government services, National Institute for Health and Welfare, Oulu, Finland

9 Department of Orthopedics, Karolinska University Hospital, Stockholm, Sweden

10 Department of Clinical Sciences, Intervention and Technology (CLINTEC), Karolinska Institutet, Stockholm, Sweden

11 Molecular Neurology Research Program, University of Helsinki, Helsinki, Finland

12 Folkhälsan Institute of Genetics, Helsinki, Finland

13 Institute for Molecular Medicine Finland, University of Helsinki, Helsinki, Finland

† These authors contributed equally to the work

* tea.kaartokallio@helsinki.fi

SUPPLEMENTARY INFORMATION

Clinical definitions

Pre-eclampsia was defined as systolic blood pressure ≥140 mmHg and/or diastolic blood pressure ≥90 mmHg with new-onset proteinuria (≥0.3g/24h) after 20 weeks of gestation[1]. Pre-eclampsia was defined early-onset when diagnosed before 34+0 weeks of gestation, and severe in the presence of systolic blood pressure ≥160 mmHg, diastolic blood pressure ≥110 mmHg, proteinuria ≥5 g/24h or clinically severe symptoms of preeclampsia, including clonus or respiratory distress. Birth weights below −2.0 SD units (birth weight relative to sex and length of gestation) according to Finnish standards[2] were classified as SGA. Placental insufficiency was defined as uterine artery resistance index or pulsatility index >+2 SD units. In the FINNPEC cohort, the diagnoses were determined based on medical records and verified independently by a research nurse and a study physician.

Sample collection and DNA extraction

A venous blood sample of 36 ml was drawn from each study participant, and genomic DNA was extracted from whole blood using the NucleoSpin Blood XL DNA extraction kit (Macherey-Nagel GmbH & Co.) or Chemagic Magnetic Separation Module I –machine (Chemagen). The extracted DNA was stored at -20°C.

Exome sequencing

Exome sequencing was performed at the Science for Life Laboratory, Stockholm, Sweden. DNA libraries were prepared from 3 µg of input material sheared to 300 bp using a Covaris S2 instrument (Covaris, MA, USA) using SureSelectXT kits (Agilent, CA, USA) and an Agilent NGS workstation according to the manufacturer's instructions (SureSelectXT Automated Target Enrichment for Illumina Paired-End Multiplexed Sequencing, version A). The SureSelectXT Human All Exon 50 Mb capture kit was used for the targeted enrichment. The clustering was performed on a cBot

cluster generation system using a HiSeq paired-end read cluster generation kit according to the manufacturer's instructions. The samples were sequenced on an Illumina HiSeq 2000 as paired-end reads to 100 bp following the manufacturer's instructions. Base conversion was performed using Illumina's OLB v1.9.

Supplementary Table S1. Phenotypes per pool in the exome sequencing

Pool	Phenotype
PE 1	Early-onset, severe pre-eclampsia
PE 2	Early-onset, severe pre-eclampsia
PE 3	Severe pre-eclampsia, proteinuria >6g/24h
PE 4	Severe pre-eclampsia, proteinuria >6g/24h
PE 5	Severe pre-eclampsia, previous miscarriage(s)
PE 6	Severe pre-eclampsia, proteinuria >3g/24h
PE 7	Severe pre-eclampsia
PE 8	Severe pre-eclampsia
PE 9	Severe, recurrent pre-eclampsia
PE 10	Familial pre-eclampsia

References

1. ACOG Committee on Obstetric Practice. Practice bulletin #33: diagnosis and management of preeclampsia and eclampsia. *Obstetrics & Gynecology* **99**, 159-167 (2002).

2. Pihkala, J., Hakala, T., Voutilainen, P. & Raivio, K. Characteristic of recent fetal growth curves in Finland. *Duodecim* **105**, 1540-1546 (1989).
